# Health anxiety, perceived stress, and coping styles in the shadow of the COVID-19

**DOI:** 10.1186/s40359-021-00560-3

**Published:** 2021-04-06

**Authors:** Szabolcs Garbóczy, Anita Szemán-Nagy, Mohamed S. Ahmad, Szilvia Harsányi, Dorottya Ocsenás, Viktor Rekenyi, Ala’a B. Al-Tammemi, László Róbert Kolozsvári

**Affiliations:** 1 Doctoral School of Health Sciences, University of Debrecen, Debrecen, Hungary; 2 Department of Psychiatry, Faculty of Medicine, University of Debrecen, Debrecen, Hungary; 3 Department of Personality and Clinical Psychology, Institute of Psychology, University of Debrecen, Debrecen, Hungary; 4 Faculty of Medicine, University of Debrecen, Debrecen, Hungary; 5 Department of Social and Work Psychology, Institute of Psychology, University of Debrecen, Debrecen, Hungary; 6 Doctoral School of Human Sciences, University of Debrecen, Debrecen, Hungary; 7 Department of Family and Occupational Medicine, Faculty of Medicine, University of Debrecen, Móricz Zs. krt. 22, Debrecen, 4032 Hungary

**Keywords:** COVID-19, Pandemic, Lockdown, Health anxiety, Perceived stress, Coping styles, Hungary, University students

## Abstract

**Background:**

In the case of people who carry an increased number of anxiety traits and maladaptive coping strategies, psychosocial stressors may further increase the level of perceived stress they experience. In our research study, we aimed to examine the levels of perceived stress and health anxiety as well as coping styles among university students amid the COVID-19 pandemic.

**Methods:**

A cross-sectional study was conducted using an online-based survey at the University of Debrecen during the official lockdown in Hungary when dormitories were closed, and teaching was conducted remotely. Our questionnaire solicited data using three assessment tools, namely, the Perceived Stress Scale (PSS), the Ways of Coping Questionnaire (WCQ), and the Short Health Anxiety Inventory (SHAI).

**Results:**

A total of 1320 students have participated in our study and 31 non-eligible responses were excluded. Among the remaining 1289 participants, 948 (73.5%) and 341 (26.5%) were Hungarian and international students, respectively. Female students predominated the overall sample with 920 participants (71.4%). In general, there was a statistically significant positive relationship between perceived stress and health anxiety. Health anxiety and perceived stress levels were significantly higher among international students compared to domestic ones. Regarding coping, wishful thinking was associated with higher levels of stress and anxiety among international students, while being a goal-oriented person acted the opposite way. Among the domestic students, cognitive restructuring as a coping strategy was associated with lower levels of stress and anxiety. Concerning health anxiety, female students (domestic and international) had significantly higher levels of health anxiety compared to males. Moreover, female students had significantly higher levels of perceived stress compared to males in the international group, however, there was no significant difference in perceived stress between males and females in the domestic group.

**Conclusion:**

The elevated perceived stress levels during major life events can be further deepened by disengagement from home (being away/abroad from country or family) and by using inadequate coping strategies. By following and adhering to the international recommendations, adopting proper coping methods, and equipping oneself with the required coping and stress management skills, the associated high levels of perceived stress and anxiety could be mitigated.

## Introduction

On March 4, 2020, the first cases of coronavirus disease were declared in Hungary. One week later, the World Health Organization (WHO) declared COVID-19 as a global pandemic [1]. The Hungarian government ordered a ban on outdoor public events with more than 500 people and indoor events with more than 100 participants to reduce contact between people [2]. On March 27, the government imposed a nationwide lockdown for two weeks effective from March 28, to mitigate the spread of the pandemic. Except for food stores, drug stores, pharmacies, and petrol stations, all other shops and educational institutions remained closed. On April 16, a week-long extension was further announced [3].

The COVID-19 pandemic with its high morbidity and mortality has already afflicted the psychological and physical wellbeing of humans worldwide [4–9]. During major life events, people may have to deal with more stress. Stress can negatively affect the population’s well-being or function when they construe the situation as stressful and they cannot handle the environmental stimuli [10]. Various inter-related and inter-linked concepts are present in such situations including stress, anxiety, and coping. According to the literature, perceived stress can lead to higher levels of anxiety and lower levels of health-related quality of life [11]. Another study found significant and consistent associations between coping strategies and the dimensions of health anxiety [12].

Health anxiety is one of the most common types of anxiety and it describes how people think and behave toward their health and how they perceive any health-related concerns or threats. Health anxiety is increasingly conceptualized as existing on a spectrum [13, 14], and as an adaptive signal that helps to develop survival-oriented behaviors. It also occurs in almost everyone’s life to a certain degree and can be rather deleterious when it is excessive [13, 14]. Illness anxiety or hypochondriasis is on the high end of the spectrum and it affects someone’s life when it interferes with daily life by making people misinterpret the somatic sensations, leading them to think that they have an underlying condition [14].

According to the American Psychiatric Association—Diagnostic and Statistical Manual of Mental Disorders (fifth edition), Illness anxiety disorder is described as a preoccupation with acquiring or having a serious illness, and it reflects the high spectrum of health anxiety [15]. Somatic symptoms are not present or if they are, then only mild in intensity. The preoccupation is disproportionate or excessive if there is a high risk of developing a medical condition (e.g., family history) or the patient has another medical condition. Excessive health-related behaviors can be observed (e.g., checking body for signs of illness) and individuals can show maladaptive avoidance as well by avoiding hospitals and doctor appointments [15].

Health anxiety is indeed an important topic as both its increase and decrease can progress to problems [14]. Looking at health anxiety as a wide spectrum, it can be high or low [16]. While people with a higher degree of worry and checking behaviors may cause some burden on healthcare facilities by visiting them too many times (e.g., frequent unnecessary visits), other individuals may not seek medical help at healthcare units to avoid catching up infections for instance. A lower degree of health anxiety can lead to low compliance with imposed regulations made to control a pandemic [17].

The COVID-19 pandemic as a major event in almost everyone’s life has posed a great impact on the population’s perceived stress level. Several studies about the relation between coping and response to epidemics in recent and previous outbreaks found higher perceived stress levels among people [18–21]. Being a woman, low income, and living with other people all were associated with higher stress levels [18]. Protective factors like being emotionally more stable, having self-control, adaptive coping strategies, and internal locus of control were also addressed [19, 20]. The findings indicated that the COVID-19 crisis is perceived as a stressful event. The perceived stress was higher amongst people than it was in situations with no emergency. Nervousness, stress, and loss of control of one’s life are the factors that are most connected to perceived stress levels which leads to the suggestion that unpredictability and uncontrollability take an important part in perceived stress during a crisis [19, 20].

Moreover, certain coping styles (e.g., having a positive attitude) were associated with less psychological distress experiences but avoidance strategies were more likely to cause higher levels of stress [21]. According to Lazarus (1999), individuals differ in their perception of stress if the stress response is viewed as the interaction between the environment and humans [22]. An Individual can experience two kinds of evaluation processes, one to appraise the external stressors and personal stake, and the other one to appraise personal resources that can be used to cope with stressors [22, 23]. If there is an imbalance between these two evaluation processes, then stress occurs, because the personal resources are not enough to cope with the stressor’s demands [23].

During stressful life events, it is important to pay attention to the increasing levels of health anxiety and to the kind of coping mechanisms that are potential factors to mitigate the effects of high anxiety. The transactional model of stress by Lazarus and Folkman (1987) provides an insight into these kinds of factors [24]. Lazarus and Folkman theorized two types of coping responses: emotion-focused coping, and problem-focused coping. Emotion-focused coping strategies (e.g., distancing, acceptance of responsibility, positive reappraisal) might be used when the source of stress is not embedded in the person’s control and these strategies aim to manage the individual’s emotional response to a threat. Also, emotion-focused coping strategies are directed at managing emotional distress [24]. On the other hand, problem-focused coping strategies (e.g., confrontive coping, seeking social support, planful problem-solving) help an individual to be able to endure and/or minimize the threat, targeting the causes of stress in practical ways [24]. It was also addressed that emotion-focused coping mechanisms were used more in situations appraised as requiring acceptance, whereas problem-focused forms of coping were used more in encounters assessed as changeable [24].

A recent study in Hunan province in China found that the most effective factor in coping with stress among medical staff was the knowledge of their family’s well-being [25]. Although there have been several studies about the mental health of hospital workers during the COVID-19 pandemic or other epidemics (e.g., SARS, MERS) [26–29], only a few studies from recent literature assessed the general population’s coping strategies. According to Gerhold (2020) [30], older people perceived a lower risk of COVID-19 than younger people. Also, women have expressed more worries about the disease than men did. Coping strategies were highly problem-focused and most of the participants reported that they listen to professionals’ advice and tried to remain calm [30]. In the same study, most responders perceived the COVID-19 pandemic as a global catastrophe that will severely affect a lot of people. On the other hand, they perceived the pandemic as a controllable risk that can be reduced. Dealing with macrosocial stressors takes faith in politics and in those people, who work with COVID-19 on the frontline.

Mental disorders are found prevalent among college students and their onset occurs mostly before entry to college [31]. The diagnosis and timely interventions at an early stage of illness are essential to improve psychosocial functioning and treatment outcomes [31]. According to research that was conducted at the University of Debrecen in Hungary a few years ago, the students were found to have high levels of stress and the rate of the participants with impacted mental health was alarming [32]. With an unprecedented stressful event like the COVID-19 crisis, changes to the mental health status of people, including students, are expected.

## Aims of the study

In our present study, we aimed at assessing the levels of health anxiety, perceived stress, and coping styles among university students amidst the COVID-19 lockdown in Hungary, using three validated assessment tools for each domain.

## Methods and materials

### Study design and setting

This study utilized a cross-sectional design, using online self-administered questionnaires that were created and designed in Google Forms® (A web-based survey tool). Data collection was carried out in the period April 30, 2020, and May 15, 2020, which represents one of the most stressful periods during the early stage of the COVID-19 pandemic in Hungary when the official curfew/lockdown was declared along with the closure of dormitories and shifting to online remote teaching. The first cases of COVID-19 were declared in Hungary on March 4, 2020. On April 30, 2020, there were 2775 confirmed cases, 312 deaths, and 581 recoveries. As of May 15, 2020, the number of confirmed cases, deaths, and recovered persons was 3417, 442, and 1287, respectively.

Our study was conducted at the University of Debrecen, which is one of the largest higher education institutions in Hungary. The University is located in the city of Debrecen, the second-largest city in Hungary. Debrecen city is considered the educational and cultural hub of Eastern Hungary. As of October 2019, around 28,593 students were enrolled in various study programs at the University of Debrecen, of whom, 6,297 were international students [33]. The university offers various degree courses in Hungarian and English languages.

### Study participants and sampling

The target population of our study was students at the University of Debrecen. Students were approached through social media platforms (e.g., Facebook®) and the official student administration system at the University of Debrecen (Neptun). The invitation link to our survey was sent to students on the web-based platforms described earlier. By using the Neptun system, we theoretically assumed that our survey questionnaire has reached all students at the University. The students who were interested and willing to participate in the study could fill out our questionnaire anonymously during the determined study period; thus, employing a convenience sampling approach. All students at the University of Debrecen whose age was 18 years or older and who were in Hungary during the outbreak had the eligibility to participate in our study whether undergraduates or postgraduates.

### Study instruments

In our present study, the survey has solicited information about the sociodemographic profile of participants including age (in years), gender (female vs male), study program (health-related vs non-health related), and whether the student stayed in Hungary or traveled abroad during the period of conducting our survey in the outbreak. Our survey has also adopted three international scales to collect data about health anxiety, coping styles, and perceived stress during the pandemic crisis. As the language of instruction for international students at the University of Debrecen is English, and English fluency is one of the criteria for international students’ admission at the University of Debrecen, the international students were asked to fill out the English version of the survey and the scales. On the other hand, the Hungarian students were asked to fill out the Hungarian version of the survey and the validated Hungarian scales. Also, we provided contact information for psychological support when needed. Students who felt that they needed some help and psychological counseling could use the contact information of our peer supporters. Four International students have used this opportunity and were referred to a higher level of care. The original scales and their validated Hungarian versions are described in the following sections.

#### Perceived Stress Scale (PSS)

The Perceived Stress Scale (PSS) measures the level of stress in the general population who have at least completed a junior high school [34]. In the PSS, the respondents had to report how often certain things occurred like nervousness; loss of control; feeling of upset; piling up difficulties that cannot be handled; or on the contrary how often the students felt they were able to handle situations; and were on top of things. For the International students, we used the 10-item PSS (English version). The statements’ responses were scored on a 5-point Likert scale (from 0 = never to 4 = very often) as per the scale’s guide. Also, in the 10-item PSS, four positive items were reversely scored (e.g. felt confident about someone’s ability to handle personal problems) [34]. The PSS has satisfactory psychometric properties with a Cronbach’s alpha of 0.78, and this English version was used for international students in our study.

For the Hungarian students, we used the Hungarian version of the PSS, which has 14 statements that cover the same aspects of stress described earlier. In this version of the PSS, the responses were evaluated on a 5-point Likert scale (0–4) to mark how typical a particular behavior was for a respondent in the last month [35]. The Hungarian version of the PSS was psychometrically validated in 2006. In the validation study, the Hungarian 14-item PSS has shown satisfactory internal consistency with a Cronbach’s alpha of 0.88 [35].

#### Ways of Coping Questionnaire (WCQ)

The second scale we used was the 26-Item Ways of Coping Questionnaire (WCQ) which was developed by Sørlie and Sexton [36]. For the international students, we used the validated English version of the 26-Item WCQ that distinguished five different factors, including *Wishful thinking* (hoped for a miracle, day-dreamed for a better time), *Goal-oriented* (tried to analyze the problem, concentrated on what to do), *Seeking support* (talked to someone, got professional help), *Thinking it over* (drew on past experiences, realized other solutions), and *Avoidance* (refused to think about it, minimized seriousness of it). The WCQ examined how often the respondents used certain coping mechanisms, eg: hoped for a miracle, fantasized, prepared for the worst, analyzed the problem, talked to someone, or on the opposite did not talk to anyone, drew conclusions from past things, came up with several solutions for a problem or contained their feelings. As per the 26-item WCQ, responses were scored on a 4-point Likert scale (from 0 = “does not apply and/or not used” to 3 = “used a great deal”). This scale has satisfactory psychometric properties with Cronbach's alpha for the factors ranged from 0.74 to 0.81[36].

For the Hungarian students, we used the Hungarian 16-Item WCQ, which was validated in 2008 [37]. In the Hungarian WCQ, four dimensions were identified, which were *cognitive restructuring/adaptation* (every cloud has a silver lining), *Stress reduction* (by eating; drinking; smoking), *Problem analysis* (I tried to analyze the problem), and *Helplessness/Passive coping* (I prayed; used drugs) [37]. The Cronbach’s alpha values for the Hungarian WCQ’s dimensions were in the range of 0.30–0.74 [37].

#### Short Health Anxiety Inventory (SHAI)

The third scale adopted was the 18-Items Short Health Anxiety Inventory (SHAI). Overall, the SHAI has two subscales. The first subscale comprised of 14 items that examined to what degree the respondents were worried about their health in the past six months; how often they noticed physical pain/ache or sensations; how worried they were about a serious illness; how much they felt at risk for a serious illness; how much attention was drawn to bodily sensations; what their environment said, how much they deal with their health. The second subscale of SHAI comprised of 4 items (negative consequences if the illness occurs) that enquired how the respondents would feel if they were diagnosed with a serious illness, whether they would be able to enjoy things; would they trust modern medicine to heal them; how many aspects of their life it would affect; how much they could preserve their dignity despite the illness [38]. One of four possible statements (scored from 0 to 3) must be chosen. Alberts et al. (2013) [39] found the mean SHAI value to be 12.41 (± 6.81) in a non-clinical sample. The original 18-item SHAI has Cronbach’s alpha values in the range of 0.74–0.96 [39]. For the Hungarian students, the Hungarian version of the SHAI was used. The Hungarian version of SHAI was validated in 2011 [40]. The scoring differs from the English version in that the four statements were scored from 1 to 4, but the statements themselves were the same. In the Hungarian validation study, it was found that the SHAI mean score in a non-clinical sample (university students) was 33.02 points (± 6.28) and the Cronbach's alpha of the test was 0.83 [40].

### Data analyses

Data were extracted from Google Forms® as an Excel sheet for quality check and coding then we used SPSS® (v.25) and RStudio statistical software packages to analyze the data. Descriptive and summary statistics were presented as appropriate. To assess the difference between groups/categories of anxiety, stress, and coping styles, we used the non-parametric Kruskal–Wallis test, since the variables did not have a normal distribution and for post hoc tests, we used the Mann–Whitney test. Also, we used Spearman’s rank correlation to assess the relationship between health anxiety and perceived stress within the international group and the Hungarian group. Comparison between international and domestic groups and different genders in terms of health anxiety and perceived stress levels were also conducted using the Mann–Whitney test. Binary logistic regression analysis was also employed to examine the associations between different coping styles/ strategies (treated as independent variables) and both, health anxiety level and perceived stress level (treated as outcome variables) using median splits. A p-value less than 5% was implemented for statistical significance.

### Ethical considerations

Ethical permission was obtained from the Hungarian Ethical Review Committee for Research in Psychology (Reference number: 2020-45). All methods were carried out following the institutional guidelines and conforming to the ethical standards of the declaration of Helsinki. All participants were informed about the study and written informed consent was obtained before completing the survey. There were no rewards/incentives for completing the survey.

## Results

### Sociodemographic characteristics of respondents

A total of 1320 students have responded to our survey. Six responses were eliminated due to incompleteness and an additional 25 responses were also excluded as the students filled out the survey from abroad (International students who were outside Hungary during the period of conducting our study). After exclusion of the described non-eligible responses (a total of 31 responses), the remaining 1289 valid responses were included in our analysis. Out of 1289 participants (100%), 73.5% were Hungarian students and around 26.5% were international students. Overall, female students have predominated the sample (n = 920, 71.4%). The median age (Interquartile range) among Hungarian students was 22 years (5) and for the international students was 22 years (4). Out of the total sample, most of the Hungarian students were enrolled in non-health-related programs (n = 690, 53.5%), while most of the international students were enrolled in health-related programs (n = 213, 16.5%). Table 1 demonstrates the sociodemographic profile of participants (Hungarian vs International).

**Table 1:** Sociodemographic characteristics of the students (n = 1289)

Variables	Hungarian (*n* = 948)	International (*n* = 341)	Chi-square test
Gender			*χ*^*2*^(1) = 35.06; *p* < 0.001
Female	719 (75.84%)	201 (58.94%)	
Male	229 (24.16%)	140 (41.06%)	
Age, median (inter quartile range)	22 (5)	22 (4)	χ^2^(1) = 3.305; *p* = 0.069
(using median split of 22)			
Faculty/study program			*χ*^*2*^ (1) = 146.21; *p* < 0.001
Health-related	258 (27.22%)	213 (62.46%)	
Non health-related	690 (72.78%)	128 (37.54%)	

### Perceived stress, anxiety, and coping styles

For greater clarity of statistical analysis and interpretation, we created preferences regarding coping mechanisms. That is, we made the categories based on which coping factor (in the international sample) or dimension (in the Hungarian sample) the given person reached the highest scores, so it can be said that it is the person's preferred coping strategy. The four coping strategies among international students were goal-oriented, thinking it over, wishful thinking, and avoidance, while among the Hungarian students were cognitive restructuring, problem analysis, stress reduction, and passive coping.

The 26-item WCQ [31] contains a seeking support subscale which is missing from the Hungarian 16-item WCQ [32]; therefore, the seeking support subscale was excluded from our analysis. Moreover, because the PSS contained a different number of items in English and Hungarian versions (10 items vs 14 items), we looked at the average score of the answers so that we could compare international and domestic students.

In the evaluation of SHAI, the scoring of the two questionnaires are different. For the sake of comparability between the two samples, the international points were corrected to the Hungarian, adding plus one to the value of each answer. This may be the reason why we obtained higher results compared to international standards.

Among the international students, the mean score (± standard deviation) of perceived stress among male students was 2.11(± 0.86) compared to female students 2.51 (± 0.78), while the mean score (± standard deviation) of health anxiety was 34.12 (± 7.88) and 36.31 (± 7.75) among males and females, respectively. Table 2 shows more details regarding the perceived stress scores and health anxiety scores stratified by coping strategies among international students.

**Table 2:** Perceived stress and health anxiety as per different coping strategies among the International students

Coping strategy	Perceived stress (PSS)		Health anxiety (SHAI)	
	Mean	SD	Mean*	SD
Goal oriented	1.82	0.73	32.05	7.55
Thinking it over	2.19	0.78	35.77	7.69
Wishful thinking	2.71	0.71	37.78	7.56
Avoidance	2.23	0.89	33.25	6.69
Overall	2.35	0.83	35.41	7.87

*SD* standard deviation

*Corrected SHAI values

In the Hungarian sample, the mean score (± standard deviation) of perceived stress among male students was 2.06 (± 0.84) compared to female students 2.18 (± 0.83), while the mean score (± standard deviation) of health anxiety was 33.40 (± 7.63) and 35.05 (± 7.39) among males and females, respectively. Table 3 shows more details regarding the perceived stress scores and health anxiety scores stratified by coping strategies among Hungarian students.

**Table 3:** Perceived stress and health anxiety depending on different coping strategies among the Hungarian students

Coping strategy	Perceived stress (PSS)		Health anxiety (SHAI)	
	Mean	SD	Mean	SD
Cognitive restructuring	1.73	0.74	32.43	6.76
Problem analysis	2.34	0.78	35.54	7.15
Stress reduction	2.79	0.64	38.36	8.06
Passive coping	2.59	0.66	37.41	8.56
Overall	2.15	0.83	34.65	7.48

*SD* standard deviation

Concerning coping styles among international students, the statements with the highest-ranked responses were “wished the situation would go away or somehow be finished” and “Had fantasies or wishes about how things might turn out” and both fall into the wishful thinking coping. Among the Hungarian students, the statements with the highest-ranked responses were “I tried to analyze the problem to understand better” (falls into problem analysis coping) and “I thought every cloud has a silver lining, I tried to perceive things cheerfully” (falls into cognitive restructuring coping).

On the other hand, the statements with the least-ranked responses among the international students belonged to the Avoidance coping. Among the Hungarians, it was Passive coping “I tried to take sedatives or medications” and Stress reduction “I staked everything upon a single cast, I started to do something risky” to have the lowest-ranked responses. Table 4 shows a comparison of different coping strategies among international and Hungarian students.

**Table 4:** Dominances of coping strategies among international and Hungarian students categorized by gender

Coping strategies	Female	Male
International students	*n* = 201	*n* = 140
Goal oriented	51 (25.37%)	44 (31.43%)
Thinking it over	23 (11.44%)	18 (12.86%)
Wishful thinking	104 (51.74%)	61 (43.57%)
Avoidance	23 (11.44%)	17 (12.14%)
Hungarian students	*n* = 719	*n* = 229
Cognitive restructuring	309 (42.98%)	99 (43.23%)
Problem analysis	279 (38.80%)	93 (40.61%)
Stress reduction	95 (13.21%)	27 (11.79%)
Passive coping	36 (5.01%)	10 (4.37%)

**Fig. 1:**
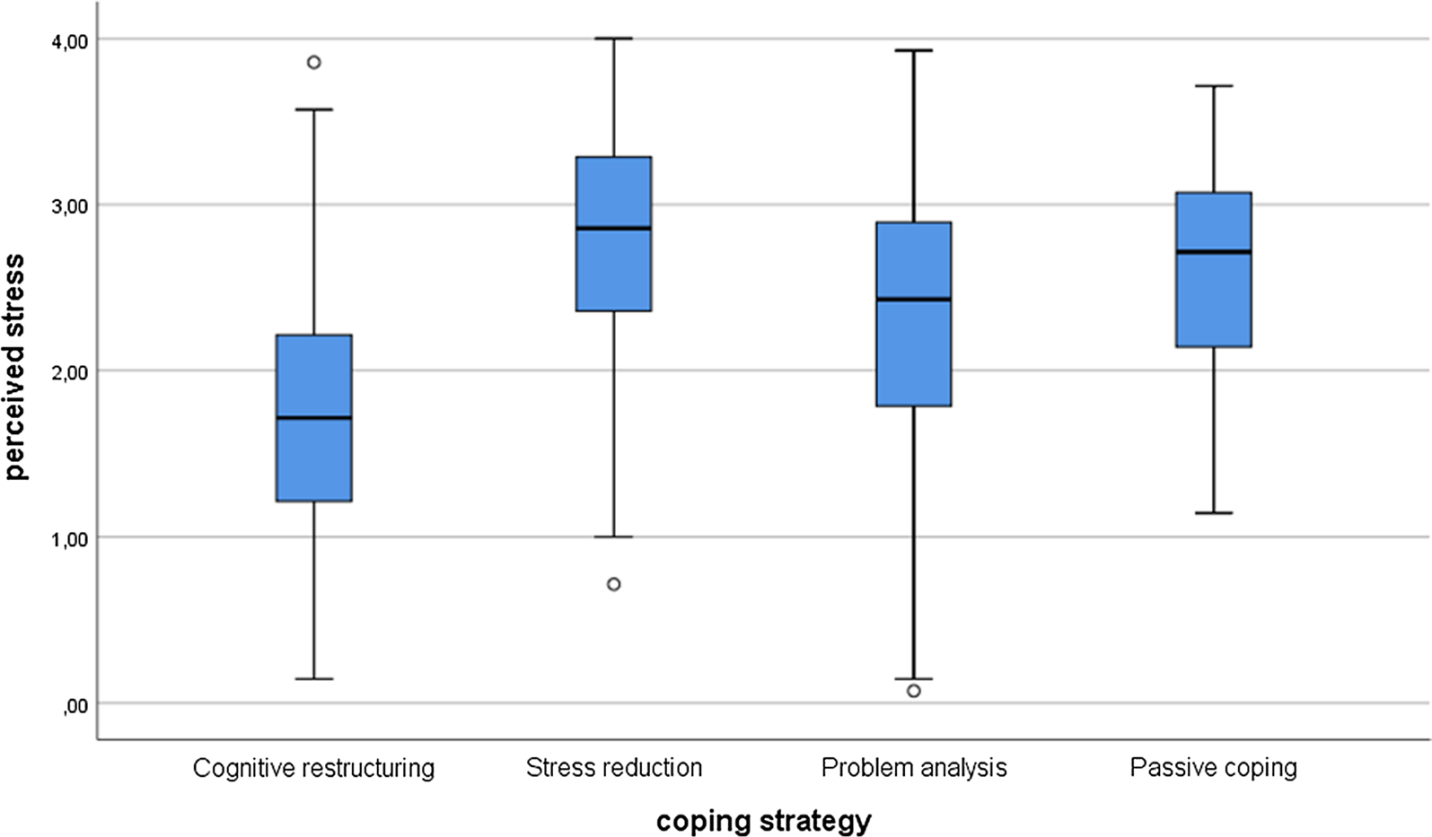
Perceived stress differences between coping strategies among the Hungarian students

**Fig. 2:**
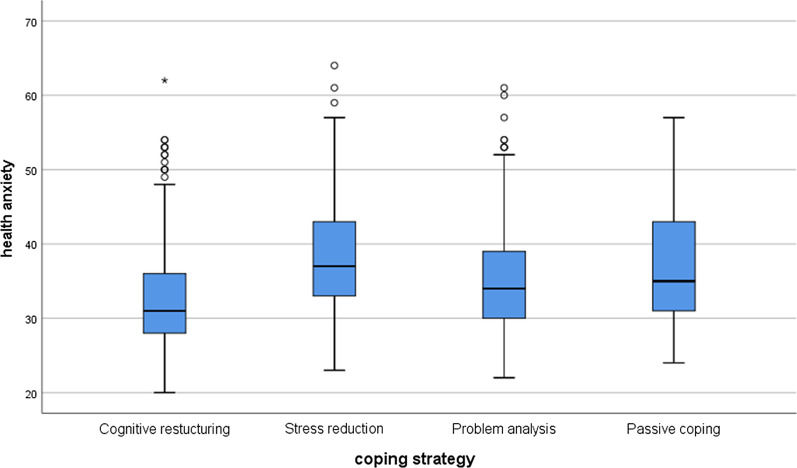
Health anxiety differences between coping strategies among the Hungarian students

To test the difference between coping strategies, we used the non-parametric Kruskal–Wallis test, since the variables did not have a normal distribution. For post hoc tests, we used Mann–Whitney tests with lowered significance levels (*p* = 0.0083). Among Hungarian students, there were significant differences between the groups in stress (*χ*^*2*^(3) = 212.01; p < 0.001) and health anxiety (*χ*^*2*^(3) = 80.32; *p* < 0.001). In the post hoc tests, there were significant differences everywhere (*p* < 0.001) except between stress reduction and passive coping (*p* = 0.089) and between problem analysis and passive coping (*p* = 0.034). Considering the health anxiety, the results were very similar. There were significant differences between all groups (*p* < 0.001), except between stress reduction and passive coping (*p* = 0.347) and between problem analysis and passive coping (*p* = 0.205). See Figs. 1 and 2 for the Hungarian students.

**Fig. 3:**
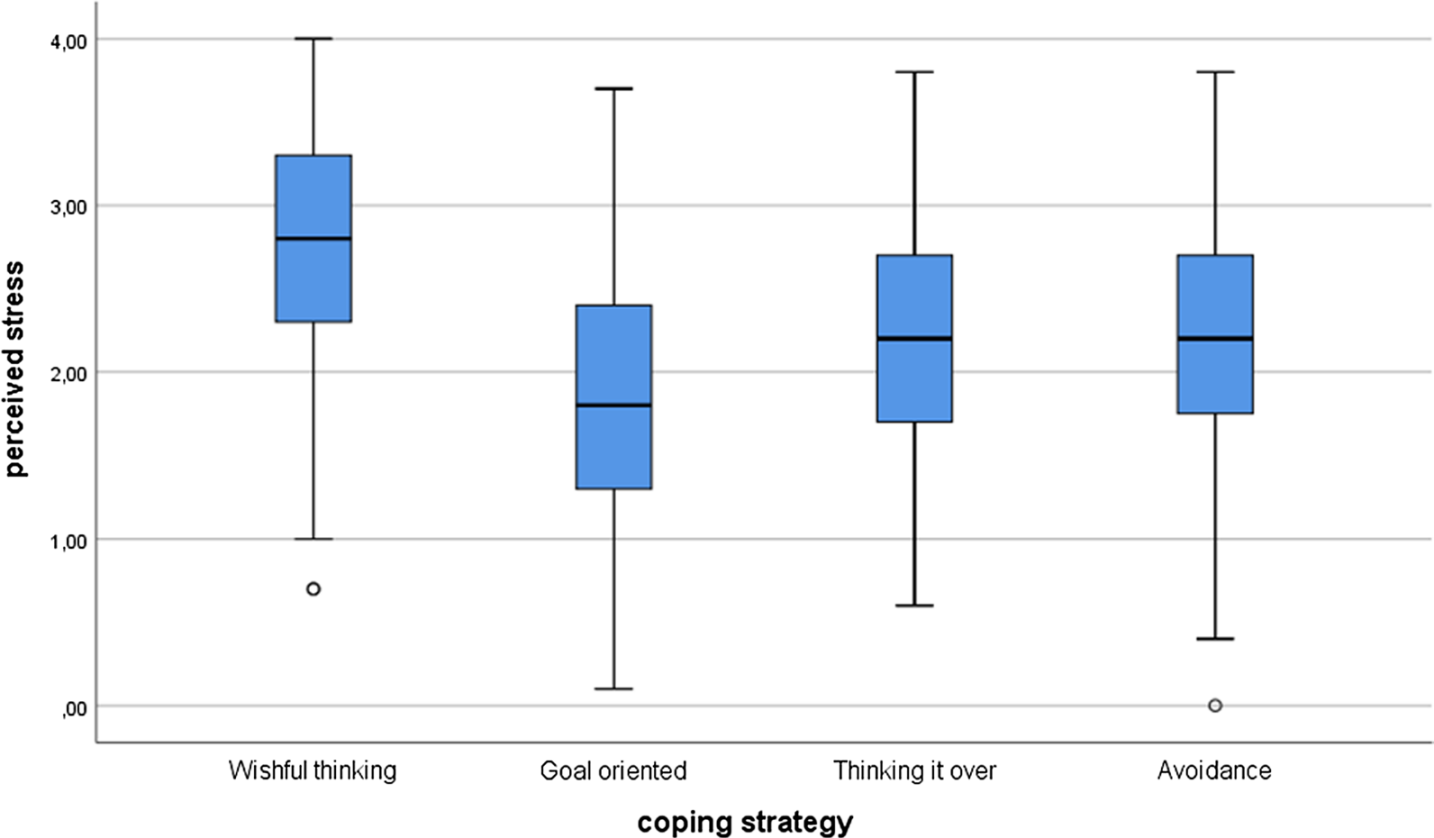
Perceived stress differences between coping strategies among the international students

**Fig. 4:**
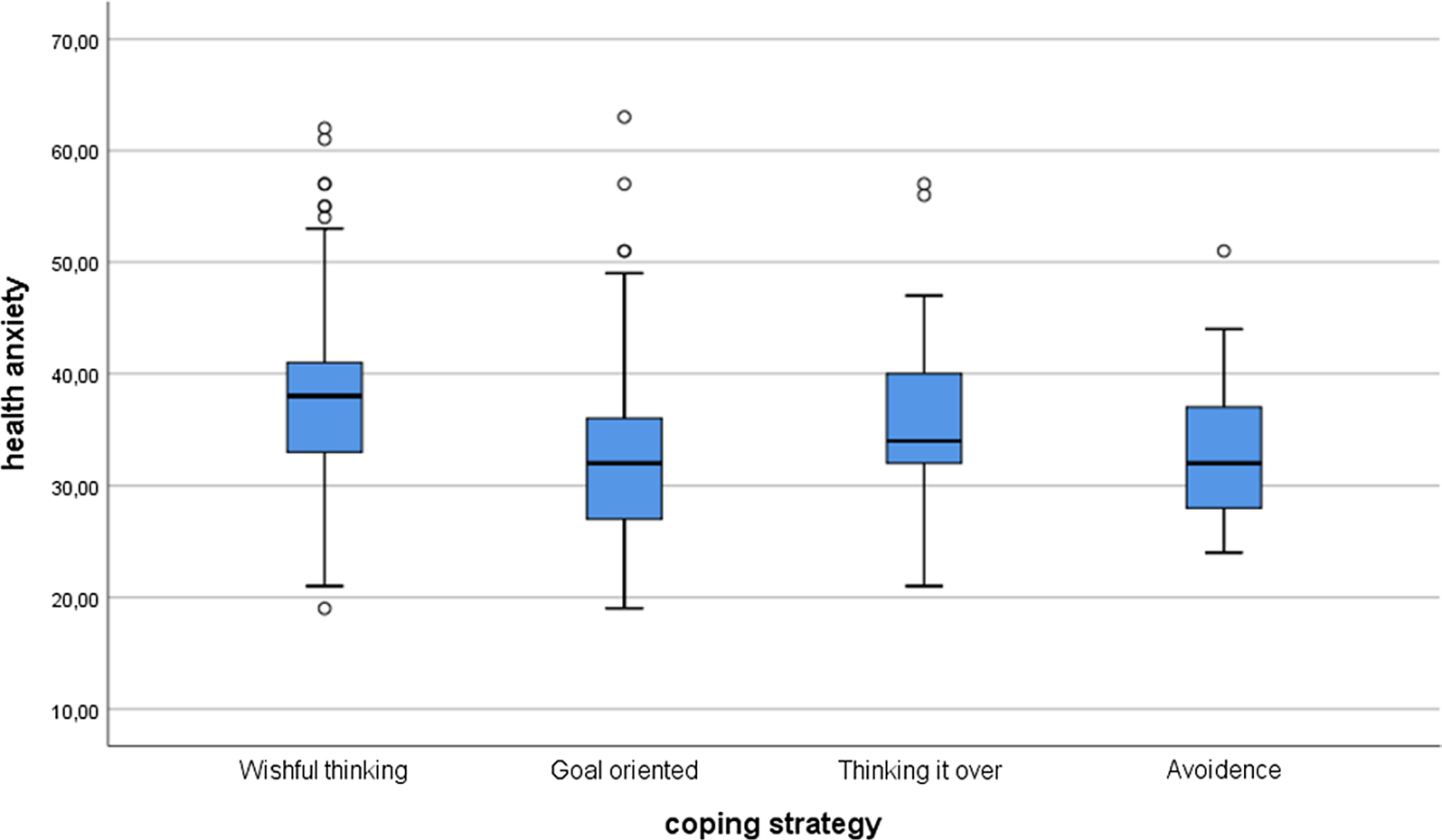
Health anxiety differences between coping strategies among the international students

Among the international students, the results were similar. According to the Kruskal–Wallis test, there were significant differences in stress (*χ*^*2*^(3) = 73.26; *p* < 0.001) and health anxiety (*χ*^*2*^(3) = 42.60; *p* < 0.001) between various coping strategies. The post hoc tests showed that there were differences between the perceived stress level and coping strategies everywhere (*p* < 0.005) except and between avoidance and thinking it over (*p* = 0.640). Concerning health anxiety, there were significant differences between wishful thinking and goal-oriented (*p* < 0.001), between wishful thinking and avoidance (*p* = 0.001), and between goal-oriented and avoidance (*p* = 0.285). There were no significant differences between wishful thinking and thinking it over (*p* = 0.069), between goal-oriented and thinking it over (*p* = 0.069), and between avoidance and thinking it over (*p* = 0.131). See Figs. 3 and 4.

### The relationship between coping strategies with health anxiety and perceived stress levels among the international students

We applied logistic regression analyses for the variables to see which of the coping strategies has a significant effect on SHAI and PSS results. In the first model (model a), with the *health anxiety* as an outcome dummy variable (with median split; median: 35), only two coping strategies had a statistically significant relationship with health anxiety level, including wishful thinking (as a risk factor) and goal-oriented (as a protective factor).

In the second model (model b), with the *perceived stress* as an outcome dummy variable (with median split; median: 2.40), three coping strategies were found to have a statistically significant association with the level of perceived stress, including wishful thinking (as a risk factor), while goal-oriented and thinking it over as protective factors. See Table 5.

**Table 5:** Logistic regression analysis for the association between coping strategies and both, the health anxiety level, and perceived stress level among international students

Dependent/outcome variables	Independent variables (coping strategy)	aOR	P-value	95% CI
Health anxiety^a^	Wishful thinking	3.24	< 0.001	2.18–4.82
	Goal-oriented	0.60	0.015	0.39–0.90
	Thinking it over	1.22	0.294	0.84–1.77
	Avoidance	1.02	0.910	0.72–1.44
Perceived stress^b^	Wishful thinking	6.74	< 0.001	4.09–11.12
	Goal-oriented	0.31	< 0.001	0.19–0.51
	Thinking it over	0.58	0.010	0.38–0.88
	Avoidance	1.23	0.282	0.84–1.80

*aOR* adjusted odds ratio, *CI* confidence interval

^a^Model (a): Health anxiety was treated as outcome dummy variable with median split; median 35. Model’s characteristics: *χ*^*2*^(1) = 16.148; *p* < 0.001; Nagelkerke *R*^*2*^ = 0.177

^b^Model (b): Perceived stress was treated as outcome dummy variable with median split; median 2,4. Model’s characteristics: *χ*^*2*^(1) = 7.179; *p* = 0.007; Nagelkerke *R*^*2*^ = 0.370

### The relationship between coping strategies with health anxiety and perceived stress levels among domestic students

By employing logistic regression analysis, with the *health anxiety* as an outcome dummy variable (with median split; median: 33.5) (model a), three coping strategies had a statistically significant relationship with health anxiety level among domestic students, including stress reduction and problem analysis (as risk factors), while cognitive restructuring (as a protective factor).

Similarly, with the *perceived stress* as an outcome dummy variable (with median split; median: 2.1429) (model b), three coping strategies had a statistically significant relationship with perceived stress level, including stress reduction and problem analysis (as risk factors), while cognitive restructuring (as a protective factor). See Table 6.

**Table 6:** Logistic regression analyses for the association between coping strategies and both, the health anxiety level, and perceived stress level among domestic students

Dependent/outcome variables	Independent variables (coping strategy)	aOR	P-value	95% CI
Health anxiety^a^	Cognitive restructuring	0.48	< 0.001	0.39–0.60
	Stress reduction	1.73	< 0.001	1.41–2.14
	Problem analysis	1.34	0.017	1.05–1.70
	Passive coping	1.12	0.133	0.95–1.43
Perceived stress^b^	Cognitive restructuring	0.27	< 0.001	0.21–0.35
	Stress reduction	2.94	< 0.001	2.31–3.73
	Problem analysis	1.87	< 0.001	1.43–2.44
	Passive coping	1.05	0.68	0.84–1.30

*aOR* adjusted odds ratio, *CI* confidence interval

^a^Model (a): Health anxiety was treated as outcome dummy variable with median split; median 33.5. Model’s characteristics: *χ*^*2*^(1) = 0.553; *p* = 0.457; Nagelkerke *R*^*2*^ = 0.116

^b^Model (b): Perceived stress was treated as outcome dummy variable with median split; median 2.1429. Model’s characteristics: *χ*^*2*^(1) = 0.494; *p* = 0.482; Nagelkerke *R*^*2*^ = 0.293

### Comparisons between domestic and international students

We compared health anxiety and perceived stress levels of the Hungarian and international students’ groups using the Mann–Whitney test. In the case of health anxiety, the results showed that there were significant differences between the two groups (*W* = 149,431; *p* = 0.038) and international students’ levels were higher. Also, there was a significant difference in the perceived stress level between the two groups (*W* = 141,024; *p* < 0.001), and the international students have increased stress levels compared to the Hungarian ones.

### Comparisons between genders within students’ groups (International vs Hungarian)

Firstly, we compared the international men’s and women’s health anxiety and stress levels using the Mann–Whitney test. The results showed that the international women’s health anxiety (*W* = 11,810; *p* = 0.012) and perceived stress (*W* = 10,371; *p* < 0.001) levels were both significantly higher than international men’s values. However, in the Hungarian sample, women’s health anxiety was significantly higher than men’s (*W* = 69,643; *p* < 0.001), but there was no significant difference in perceived stress levels among between Hungarian women and men (*W* = 75,644.5; *p* = 0.064).

### Relationship between health anxiety and perceived stress

We correlated the general health anxiety and perceived stress using Spearman’s rank correlation. There was a significant moderate positive relationship between the two variables (*p* < 0.001; *ρ* = 0.446). Within the Hungarian students, there was a significant correlation between health anxiety and perceived stress (*p* < 0.001; *ρ* = 0.433), similarly among international students as well (*p* < 0.001; *ρ* = 0.465).

## Discussion

In our study, we found that individuals who were characterized by a preference for certain coping strategies reported significantly higher perceived stress and/or health anxiety than those who used other coping methods. These correlations can be found in both the Hungarian and international students. In the light of our results, we can say that 48.4% of the international students used wishful thinking as their preferred coping method while around 43% of the Hungarian students used primarily cognitive restructuring to overcome their problems.

Regulation of emotion refers to “the processes whereby individuals monitor, evaluate, and modify their emotions in an effort to control which emotions they have, when they have them, and how they experience and express those emotions” [41]. There is an overlap between emotion-focused coping and emotion regulation strategies, but there are also differences. The overlap between the two concepts can be noticed in the fact that emotion-focused coping strategies have an emotional regulatory role, and emotion regulation strategies may “tax the individual’s resources” as the emotion-focused coping strategies do [23, 42]. However, in emotion-focused coping strategies, non-emotional tools can also be used to achieve non-emotional goals, while emotion regulation strategies may be used for maintaining or reinforcing positive emotions [42].

Based on the cognitive-behavioral model of health anxiety, emotion-regulating strategies can regulate the physiological, cognitive, and behavioral consequences of a fear response to some degree, even when the person encounters the conditioned stimulus again [12, 43]. In the long run, regular use of these dysfunctional emotion control strategies may manifest as functional impairment, which may be associated with anxiety disorders. A detailed study that examined health anxiety in the view of the cognitive-behavioral model found that, regardless of the effect of depression, there are significant and consistent correlations between certain dimensions of health anxiety and dysfunctional coping and emotional regulation strategies [12].

Similar to our current study, other studies have found that health anxiety was positively correlated with maladaptive emotion regulation and negatively with adaptive emotion regulation [44], and in the case of state anxiety that emotion-focused coping strategies proved to be less effective in reducing stress, while active coping leads to a sense of subjective well-being [17, 27, 45–47]

SHAI values were found to be high in other studies during the pandemic, and the SHAI results of the international students in our study were found to be even slightly higher compared to those studies [44, 48]. Besides, anxiety values for women were found to be higher than for men in several studies [44, 48–50]. This was similar to what we found among the international students but not among the Hungarian ones. We can speculate that the ability to contact someone, the closeness of family and beloved ones, familiarity with the living environment, and maybe less online search about the coronavirus news could be factors counting towards that finding among Hungarian students. Also, most international students were enrolled in health-related study programs and his might have affected how they perceived stress/anxiety and their preferred coping strategies as well. Literature found that students of medical disciplines could have obstacles in achieving a healthy coping strategy to deal with stress and anxiety despite their profound medical knowledge compared to non-health-related students [51, 52]. Literature also stressed the immense need for training programs to help students of medical disciplines in adopting coping skills and stress-reducing strategies [51].

The findings of our study may be a starting point for the exploration of the linkage between perceived stress, health anxiety, and coping strategies when people are not in their domestic context. People who are away from their home and friends in a relatively alien environment may tend to use coping mechanisms other than the adequate ones, which in turn can lead to increased levels of perceived stress.

Furthermore, our results seem to support the knowledge that deep-rooted health anxiety is difficult to change because it is closely related to certain coping mechanisms. It was also addressed in the literature that personality traits may have a significant influence on the coping strategy used by a person [53], revealing sophisticated and challenging links to be considered especially during training programs on effective coping and management skills. On the other hand, perceived stress which has risen significantly above the average level in the current pandemic, can be most effectively targeted by the well-formulated recommendations and advice of major international health organizations if people successfully adhere to them (e.g. physical activity; proper and adequate sleep; healthy eating; avoiding alcohol; meditation; caring for others; relationships maintenance, and using credible information resources about the pandemic, etc.) [1, 54]. Furthermore, there may be additional positive effects of these recommendations when published in different languages or languages that are spoken by a wide range of nationalities. Besides, cognitive behavioral therapy techniques, some of which are available online during the current pandemic crisis, can further reduce anxiety. Also, if someone does not feel safe or fear prevails, there are helplines to get in touch with professionals, and this applies to the University of Debrecen in Hungary, and to a certain extent internationally.

Naturally, our study had certain limitations that should be acknowledged and considered. The temporality of events could not be assessed as we employed a cross-sectional study design, that is, we did not have information on the previous conditions of the participants which means that it is possible that some of these conditions existed in the past, while others de facto occurred with COVID-19 crisis. The survey questionnaires were completed by those who felt interested and involved, i.e., a convenience sampling technique was used, this impairs the representativeness of the sample (in terms of sociodemographic variables) and the generalizability of our results. Also, the type of recruitment (including social media) as well as the online nature of the study, probably appealed more to people with an affinity with this kind of instrument. Besides, each questionnaire represented self-reported states; thus, over-reporting or under-reporting could be present. It is also important to note that international students were answering the survey questionnaire in a language that might not have been their mother language. Nevertheless, English fluency is a prerequisite to enroll in a study program at the University of Debrecen for international students. As the options for gender were only male/female in our survey questionnaire, we might have missed the views of students who do not identify themselves according to these gender categories. Also, no data on medical history/current medical status were collected. Lastly, we had to make minor changes to the used scales in the different languages for comparability.

## Conclusion

The COVID-19 pandemic crisis has imposed a significant burden on the physical and psychological wellbeing of humans. Crises like the current pandemic can trigger unprecedented emotional and behavioral responses among individuals to adapt or cope with the situation. The elevated perceived stress levels during major life events can be further deepened by disengagement from home and by using inadequate coping strategies. By following and adhering to the international recommendations, adopting proper coping strategies, and equipping oneself with the required coping and stress management skills, the associated high levels of perceived stress and anxiety might be mitigated.

## Data Availability

The datasets generated and/or analyzed during the current study are not publicly available due to compliance with institutional guidelines but they are available from the corresponding author (LRK) on a reasonable request.

## Abbreviations

CDC: Centers for Disease Control and Prevention
COVID-19: Coronavirus Disease 2019
PSS: Perceived Stress Scale
SHAI: Short Health Anxiety Inventory
MERS: Middle East Respiratory Syndrome
SARS: Severe Acute Respiratory Syndrome
WCQ: Ways of Coping Questionnaire
WHO: World Health Organization
